# Heparin Oligosaccharides Have Antiarrhythmic Effect by Accelerating the Sodium-Calcium Exchanger

**DOI:** 10.3389/fcvm.2018.00067

**Published:** 2018-06-07

**Authors:** Carlos M. G. de Godoy, Ênio R. Vasques, Afonso Caricati-Neto, José G. P. Tavares, Beatriz J. Alves, Juliana Duarte, Regiane Miranda-Ferreira, Marcelo A. Lima, Helena B. Nader, Ivarne L. dos Santos Tersariol

**Affiliations:** ^1^Institute of Science and Technology, Universidade Federal de São Paulo, São Paulo, Brazil; ^2^Department of Gastroenterology (LIM 37), Medical School, University of São Paulo, São Paulo, Brazil; ^3^Núcleo de Pesquisas Tecnológicas, Universidade de Mogi das Cruzes, Mogi das Cruzes, Brazil; ^4^Department of Pharmacology, Universidade Federal de São Paulo, São Paulo, Brazil; ^5^Department of Biochemistry, Universidade Federal de São Paulo, São Paulo, Brazil; ^6^Centro Interdisciplinar de Investigação Bioquímica, Universidade de Mogi das Cruzes, Mogi das Cruzes, Brazil

**Keywords:** arrhythmia, low molecular weight heparin, trisulfated heparin disaccharide, sodium-calcium exchanger, calcium overload

## Abstract

**Background:** Blockage of the Na^+^/Ca^2+^ exchanger (NCX) is used to determine the role of NCX in arrhythmogenesis. Trisulfated heparin disaccharide (TD) and Low Molecular Weight Heparins (LMWHs) can directly interact with the NCX and accelerate its activity.

**Objective:** In this work, we investigated the antiarrhythmic effect of heparin oligosaccharides related to the NCX activity.

**Methods:** The effects of heparin oligosaccharides were tested on the NCX current (patch clamping) and intracellular calcium transient in rat cardiomyocytes. The effects of heparin oligosaccharides were further investigated in arrhythmia induced in isolated rat atria and rats *in vivo*.

**Results:** The intracellular Ca^2+^ concentration decreases upon treatment with either enoxaparin or ardeparin. These drugs abolished arrhythmia induction in isolated atria. The NCX antagonist KB-R7943 abolished the enoxaparin or ardeparin antiarrhythmic effects in isolated atria. In the *in vivo* measurements, injection of TD 15 min both before coronary occlusion or immediately after reperfusion, significantly prevented the occurrence of reperfusion-induced arrhythmias (ventricular arrhythmia and total AV block) and reduced the lethality rate. The patch clamping experiments showed that, mechanistically, TD increases the forward mode NCX current.

**Conclusion:** Together, the data shows that heparin oligosaccharides may constitute a new class of antiarrhythmic drug that acts by accelerating the forward mode NCX under calcium overload.

## Introduction

Many authors have studied the potential antiarrhythmic effect of NCX blockers by targeting the role of the NCX in arrhythmogenesis (1, 2). Theoretically, the undesirable effects of blocking Ca^2+^ efflux on NCX activity could be limited by the predominant inhibition of its reverse mode over the forward mode. KBR-7943 was the first drug from a generation of NCX blockers with reported mode selectivity. Such drug was eventually followed by SEA-0400 (3–5), which is more potent and more selective for NCX and, most recently, by SN-6 (6) and ORM-10962 (7). Nonetheless, occasionally these drugs are not useful for NCX function studies, but they may be appropriate as part of an antiarrhythmic strategy (6, 7).

As mentioned, blockage of NCX is a common tool used to target the role of NCX in arrhythmogenesis. On the other hand, it has been shown that heparin disaccharides accelerate NCX instead of blocking it which opens new perspectives to evaluate arrhythmogenesis or other Ca^2+^ overload situations. Shinjo and colleagues observed that cumulative doses of trisulfated heparin disaccharide (TD) decrease cytosolic Ca^2+^ levels in smooth muscle cell lines derived from the rabbit aorta (8). They have also shown that heparin oligosaccharides directly interact with the NCX and accelerate the NCX activity. Recently, it was shown that TD decreases liver cell damage and increases hepatic tolerance to ischemia/reperfusion injury by calcium extrusion in Ca^2+^ overload conditions (9).

In the present work, we investigated how heparin oligosaccharides would affect arrhythmogenesis in isolated rat atria and rats *in vivo*, and NCX currents and intracellular calcium in isolated cardiomyocytes. Here, we show for the first time that Low Molecular Weight Heparins (LMWHs) and TD have significant antiarrhythmic activity mediated by the acceleration of the NCX forward mode activity.

## Methods

### Isolated atrium

The isolated rat atrium preparation and the electric field stimulation (EFS) induced tachyarrhythmia followed what was previously described (10). The isolated heart was placed in a vial with a Krebs-Henseleit solution (KH perfusate) to allow blood pumping and right atrium isolation. The KH perfusate had the following composition: 126.4 mM NaCl, 4.6 mM KCl, 1.2 mM KH_2_PO, 1.2 mM MgSO_4_, 13.6 NaHCO_3_, 1.5 mM CaCl_2_, 11.11 mM glucose, pH 7.4 at 36.5°C, saturated with 95% O_2_ plus 5% CO_2_.

The EFS protocol (rapid pacing - 250 biphasic voltage pulses of 66.7 Hz; 5 ms duration; 2-fold threshold strength) and electrogram detection of isolated atria were performed using an electric stimulator (S48–Stimulator, Grass; Inst. Division, Astro-Med Inc; W. Warwick, RI, USA) and an amplifier (Iso-Dam8; World Precision Instruments, Inc., Sarasota, FL, USA). Both devices were connected to a commercial acquisition system (AqDados® 7.02; Lynx Tecn. Electr. Ltda., São Paulo, SP, Brazil).

Arrhythmia inductions were performed in isolated right atria removed either from rats without any pharmacological treatment or from rats previously treated (1 h) with an intraperitoneal injection of enoxaparin at doses of either 0.7 or 5.7 mg/kg. The effects of 50 μM KB-R7943 (an inhibitor of the reverse NCX) and/or 100 μM enoxaparin (or ardeparin) were evaluated on EFS-induced tachyarrhythmia in the isolated right atria. Tachyarrhythmia induction by rapid pacing was performed 30–40 min after drug treatment in KH perfusate. These drug studies were performed 30 min after atrial tachyarrhythmia induced by rapid pacing and resumed to the normal rate (control); the arrhythmias sustained for at least 5 min.

### *In vivo* experiments (ischemia-reperfusion method)

A control group of rats (treated with saline solution only) was initially tested to identify any lethality in all types of arrhythmias induced by the ischemia-reperfusion procedure. Trisulfated heparin disaccharide (TD), O-(4-deoxy-hex-4-enopyranosyluronic acid 2-sulfate) -(1 → 4)-2-sulfamino-D-glucose 6-sulfate was administered intravenously (IV; 0.025 mg/kg). TD was prepared as previously described (8). Its purity and structure integrity was evaluated by Nuclear Magnetic Resonance spectroscopy.

**Scheme 1:**
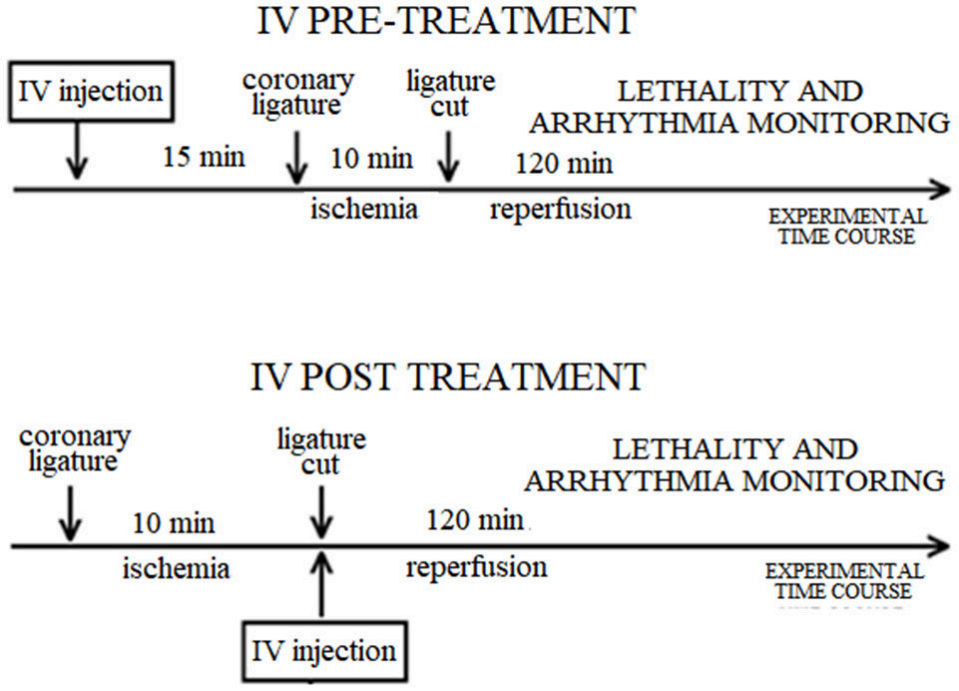
Diagram of the experimental protocol for each route of drug administration in the rat. IV: intravenous.

The experimental protocol for each route of drug administration is illustrated in Scheme 1. The ECG was recorded throughout the entire procedure, including prior to any injection.

### *In vivo* ischemia-reperfusion method for arrhythmia induction

The animal preparation followed the methods described by Guarini and colleagues (11). Rats anesthetized with urethane (1.25 g/kg IP) were immobilized in the supine position. Polyethylene catheters were inserted into a common carotid artery and a femoral vein. The arterial catheter was connected to a transducer for the recording of the mean arterial pressure on a microcomputer acquisition system (same setup described in the “Isolated Atrium” item). Drug administration was performed via the venous catheter. After opening the rat chests by left thoracotomy, the heart was exteriorized by pressure on the abdomen. A ligature was made close to the origin of the left coronary artery using silk suture. The ends of the silk suture used in the ligature were passed through a polyethylene tube. Then, the heart was in the chest cavity such that the ligature ends were exteriorized. Reperfusion was performed after 10 min of coronary occlusion by cutting the suture externally to the polyethylene tube. The animals were then further monitored during 2 h for evaluation of lethality and the occurrence of arrhythmias.

The heart rate and incidence of post ischemia-reperfusion-induced arrhythmias such as ventricular fibrillation (VF) and ventricular tachycardia (VT) were evaluated at control condition (no drug) or at presence of Trisulfated heparin disaccharide (TD), O-(4-deoxy-hex-4-enopyranosyluronic acid 2-sulfate) -(1 → 4)-2-sulfamino-D-glucose 6-sulfate. TD was administered intravenously (0.025 mg/kg) 5 min prior ischemia (pre-treatment) or in the beginning of reperfusion (post-treatment). The ECGs measurements were performed according to the Lambeth Conventions (12).

The control group (no drug treatment) was compared to the drug-treated groups using ANOVA. The incidence of arrhythmias, such as ventricular or atrial fibrillation, as well as ventricular tachyarrhythmia, was compared with Fisher's Exact Test in all groups.

All animal procedures conform to the Guide for the Care and Use do Laboratory Animals, and were approved by The Committee of Ethical in Animal Research of either University of Mogi das Cruzes - UMC (isolated rat atrium) or Federal University of São Paulo–UNIFESP (*in vivo* rats).

### Patch clamp experiments

The patch clamp protocol was designed for the recording of Na^+^/Ca^2+^ exchange current (NCX current) from isolated rat ventricular myocytes. The recordings occurred in the absence or presence of different TD concentrations. Adult rat myocytes were obtained from ventricles. The patch clamp experiments were performed at Zenas Technologies LLC (1441 Canal St. New Orleans, LA 70112).

### Current measurements and analysis

Currents were measured using the whole-cell variant of the patch clamp method. Experiments were performed at 24 ± 1°C. Glass pipettes were pulled from borosilicate glass by a horizontal puller (Sutter Instruments, USA), then fire polished to produce tip openings of 1–2 μm. An Axopatch 1B amplifier (Axon Instruments, Foster City, CA) was used for whole-cell voltage clamping. The voltage clamp pulses and data acquisition were controlled by a computer running pClamp software (ver 9.2 Axon Instruments). After rupture of the cell membrane (entering whole-cell mode), current amplitude and kinetics were allowed to stabilize (3–5 min) before starting the experiments. The voltage clamp protocol for NCX current consisted of slow-ramp pulses applied from −120 to +80 mV at 0.09 V/s and 0.1 Hz. The current which was sensitive to 5 mM Ni^2+^ was taken as the NCX current. The NCX current was obtained by subtracting the currents recorded in perfusate with Ni^2+^ (NCX blocker NiCl_2_ added at the end of each experiment) from the currents recorded in Ni^2+^-free perfusate. The holding potential was −40 mV to block T-type Ca^2+^ and Na^+^ currents. Three different concentrations of intracellular Ca^2+^ (Ca_i_; in nM: 300, 400, 600) were used in separate experiments. One drug concentration (TD: 10, 30, or 100 μM) was applied per cell (*n* = 3). The perfusate solution (compounds from Sigma-Aldrich Chemical Company) contained (in mM): NaCl 135, CsCl 10, MgCl_2_ 1, CaCl_2_ 2, HEPES 10, Dextrose 10; pH 7.4 adjusted using Cs-OH. The solution was K^+^-free to block inward rectifier K^+^ and Na^+^-K^+^-ATPase currents. The solution also contained (in μM/L): nifedipine 0.5, nifumic acid 100, ouabain 10 and 0.001 thapsigargin to block L-type Ca^2+^, Cl^−^, Na^+^-K^+^-ATPase and sarco/endoplasmic reticulum Ca^2+^ ATPase (SERCA) currents, respectively. The internal solution (pipette) contained (in mmol/L): CsCl 136, NaCl 10, Aspartate 42, MgCl_2_ 3, HEPES 5, TEA-Cl 20, MgATP 10; pH of 7.2 adjusted using Cs-OH and CaCl_2_ adjusted with BAPTA. Raw data and mean ± SEM are given. Data is presented as % change of current amplitude. This was measured as peak current increase in the presence of the tested compound relative to the current amplitude before treatment (control). Each cell served as its own control.

### Calcium transient measurements

The fluorometric analysis of cytosolic Ca^2+^ levels in rat cardiomyocytes was performed according to the method proposed by Lopes and colleagues (13). The effects of enoxaparin (Clexane®;, Sanofi-Aventis) and ardeparin (Normophilo®;, Wyeth-Ayerst Laboratories) in intracellular Ca^2+^ transient were studied in a suspension of cardiomyocytes stimulated with drugs capable of either increasing Ca^2+^ influx through the direct activation of L-type VDCC (Bay K 8644) or promoting the release of Ca^2+^ from the sarco/endoplasmic reticulum through the activation of SERCA by either ryanodine or caffeine (14). In assays with Bay K 8644, nifedipine, the selective blocker of L-type VDCC, was used to inhibit the influx of Ca^2+^ mediated by these channels. In assays with ryanodine and caffeine, a selective blocker of the SERCA, was used to inhibit the uptake of Ca^2+^ by SERCA.

## Results

### Isolated atrium

**Figure 1:**
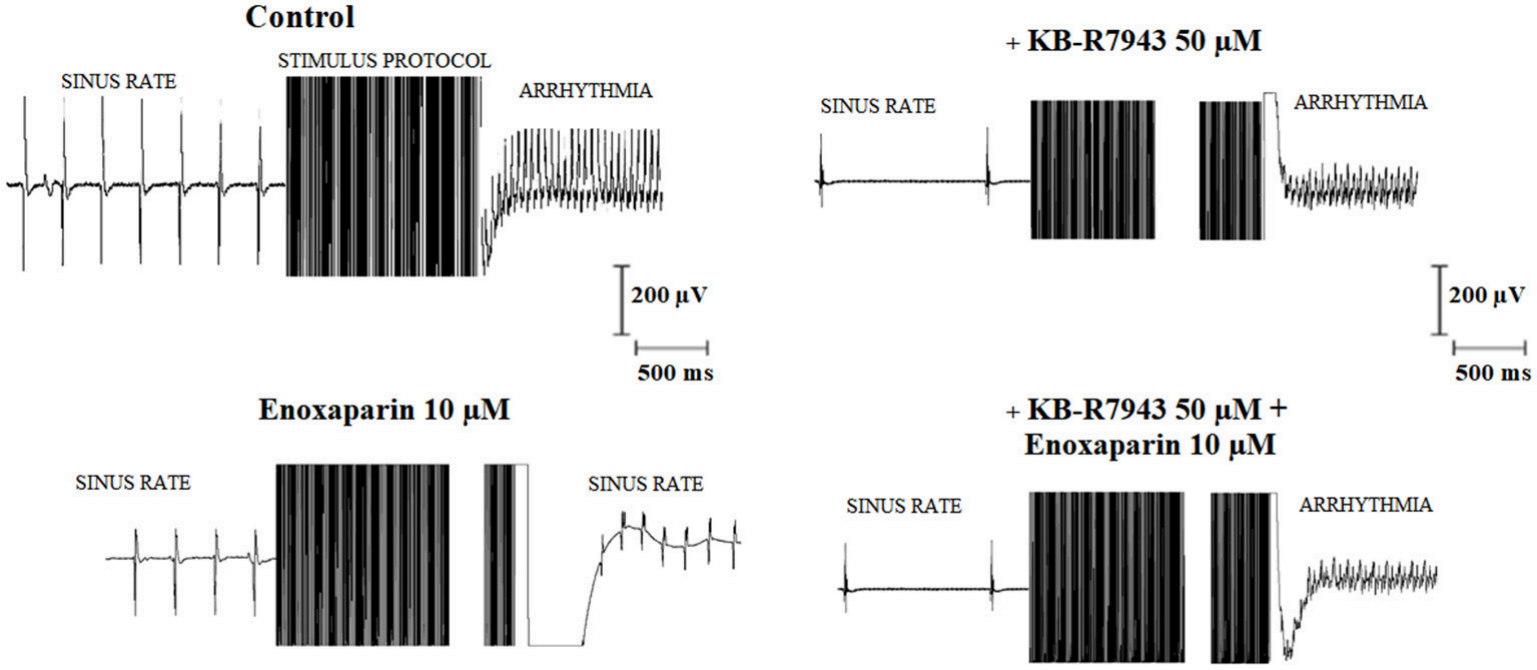
Typical electrogram records of isolated rat atria submitted to the electric field stimulation (EFS) protocol. The effect of the EFS-protocol on atrial response was studied in the absence of any drug treatment (control, **Left upper**) as well as in the presence of 50 μM KB-R7943 alone **(Right upper)**, 10 μM enoxaparin alone **(Left lower)** and the simultaneous presence of 50 μM KB-R7943 and 10 μM enoxaparin **(Right lower)**. The sinus rates (Hz) prior to the EFS-protocol were 3.7, 3.6, 0.9, and 1.2 in control, enoxaparin alone, KB-R7943 alone and enoxaparin + KB-R 7043 groups, respectively. Accordingly, the atrial rates (Hz) following the EFS-protocol for these groups were 25, 5.8, 25, and 25, respectively.

Figure 1 shows typical electrogram records of the EFS-protocol inducing arrhythmia in the right atrium (left upper panel) and enoxaparin (10 μM) preventing arrhythmia induction (left lower panel; same atrium). KB-R7943 treatment (50 μM) failed to prevent arrhythmia induction in the right atrium after EFS-protocol (right upper panel). The antiarrhythmic effect of enoxaparin was not observed after EFS-protocol application when KB-R7943 was present (right lower panel). As compared to the control, the sinus rate prior to the EFS-protocol was decreased in the presence of 50 μM KB-R7943.

LMWHs (enoxaparin, *N* = 20; ardeparin, *N* = 20) abolished atrial arrhythmia induction in all tests. Neither unfractionated heparin (N = 6) nor KB-R7943 (*N* = 6) abolished atrial arrhythmia in all tests. Pre-treatment with KB-R7943 abolished the antiarrhythmic effect of enoxaparin (*N* = 6) and ardeparin (*N* = 5), suggesting that the antiarrhythmic effect of these LMWHs requires functional NCX.

The intraperitoneal injection of enoxaparin in rats resulted in an antiarrhythmic effect in the isolated atrium. The arrhythmia induction rates (number of atria in which arrhythmia was induced/total number of atria) for the control condition and enoxaparin doses of 0.7 mg/kg and 5.7 mg/kg were 30/30, 0/5, and 0/5, respectively. The arrhythmia induction rates in the presence of enoxaparin were significantly different from that of the control condition (*p* < 0.05; *X*^2^-test).

### Animal studies

Table 1 summarizes the effect of intravenous (IV) pre-treatment with TD and IV post-treatment with TD on arrhythmias induced by post-ischemia reperfusion.

**Table 1:** Summary of ischemia/reperfusion data.

**Groups**	***N***	**Ventricular Arrhythmia**	**Total AV-Block**	**Death**
Control	33	28/33^a^	26/33^a^	23/33^a^
Pre-treatment (TD 0.025 mg/kg)	10	3/10^*^ (*P* = 0.0020)	1/10^*^ (*P* = 0.0002)	0/10^*^ (*P* < 0.0001)
Post-treatment (TD 0.025 mg/kg)	10	3/10^*^ (*P* = 0.0020)	1/10^*^ (*P* = 0.0002)	0/10^*^ (*P* < 0.0001)

a *The numbers in columns 3, 4, and 5 represent the proportions of arrhythmia or death events in relation to total number of rats in a given group*.

* *Data were considered to be statistically significant by Fisher's Exact Test (The two-sided P-values < 0.05)*.

Coronary reperfusion following a 10 min occlusion caused the occurrence or worsening of ventricular arrhythmias (ventricular extrasystoles, ventricular tachycardia, ventricular fibrillation, and Torsades de Pointes) in 28 out of 33 (85%) control rats and total AV-blocking in 26 out of 33 (79%) control rats. The lethality under such conditions was high (23/33, 70%).

Treatment with the TD (0.025 mg/kg; IV) 15 min prior coronary occlusion significantly prevented reperfusion-induced arrhythmias (ventricular arrhythmias and total AV-blocking) and reduced the lethality rate. Injection of TD (0.025 mg/kg; IV) at the early stages of reperfusion significantly prevented the occurrence of reperfusion-induced arrhythmias (ventricular arrhythmias and total AV-blocking) and reduced the lethality rate. Hence, the heparin trisulfated disaccharide is the minimum heparin fragment that is able to prevent severe arrhythmias and decrease the lethality from cardiac arrhythmias in rats.

### Effect of TD pre-treatment in ECG intervals

Pre-treatment with TD (0.025 mg/kg; IV) did not affect the normal ECG parameters of the control animals (Table 2). The normal PR intervals were unchanged by TD pre-treatment, suggesting that TD not disturb normal A-V conduction. The normal QT intervals were also unchanged by TD pre-treatment, suggesting that TD not disturb the normal time of ventricular repolarization. Finally, TD pre-treatment did not change the normal RR intervals, further suggesting that TD not alter normal heart rate.

**Table 2:** Summary of ECG intervals data.

**Group**	***N***	**ECG INTERVAL (ms)**		
		**RR**	**PR**	**QT**
Control	30	189 ± 6	52 ± 2	70 ± 1
TD 0.025 mg/kg	10	203 ± 13(*P* = 0.2278)	51 ± 1 (*P* = 0.1189)	71 ± 1 (*P* = 0.1996)

*ECG intervals are expressed as the means ± standard error. ECG intervals (either RR or PP or QT) from the control group were not significantly different from those of the TD Group (ANOVA; The two-sided P-values > 0.05)*.

### Calcium transients

**Figure 2:**
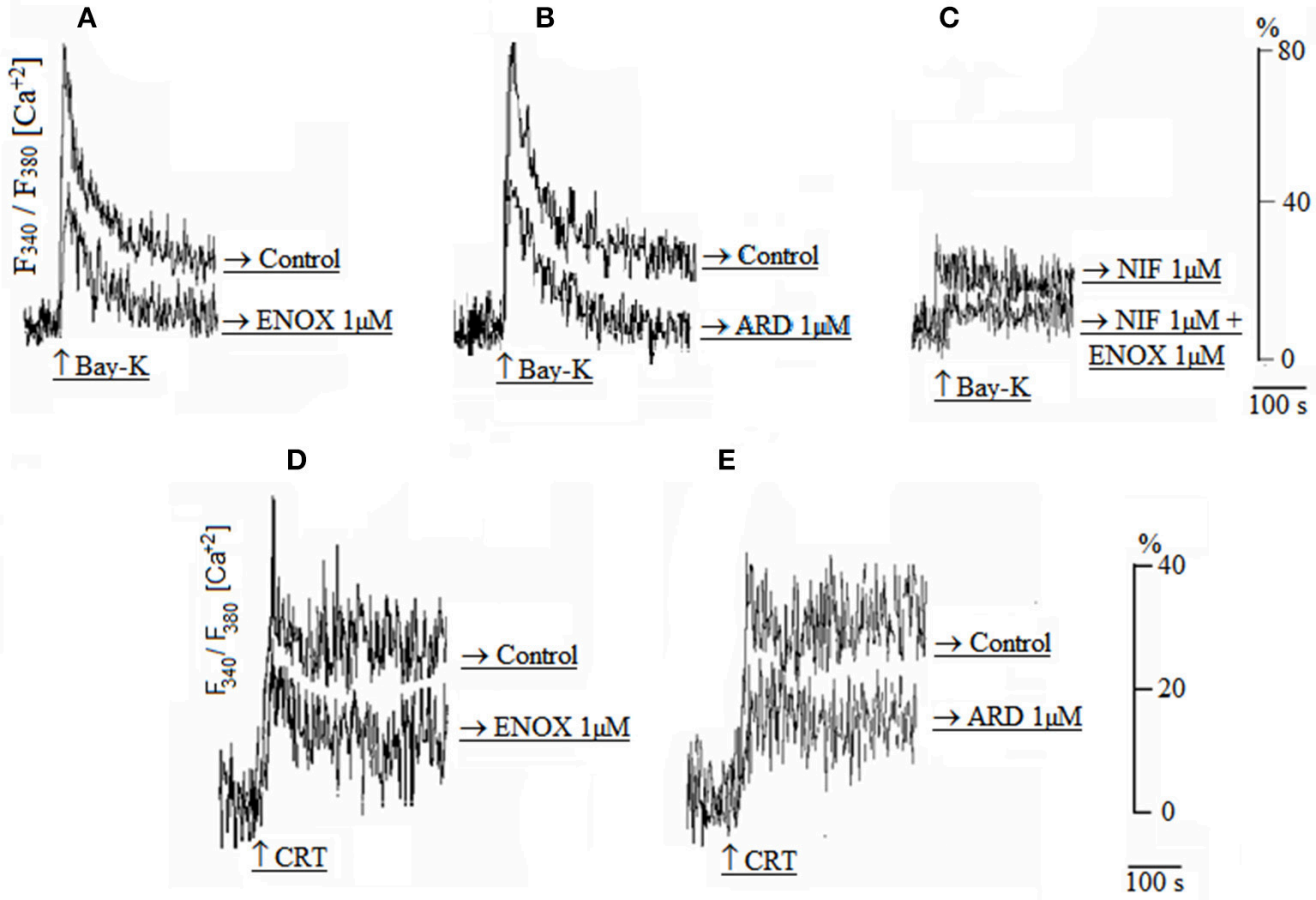
**(A,B)** Calcium transients induced by Bay-K in either the absence (control) or presence of enoxaparin (ENOX 1 μM) or ardeparin (ARD 1 μM). **(C)** Calcium transient induced by Bay-K in the presence of either nifedipine alone (NIF 1 μM) or simultaneous presence of 1 μM NIF and 1 μM ENOX. **(D,E)** Calcium transients induced by the caffeine-ryanodine-thapsgargin cocktail (CRT) in either the absence (control) or presence of 1 μM ENOX or 1 μM ARD.

As shown in Figure 2, Ca^2+^ transients induced by Bay-K was decreased by enoxaparin (Figure 2A) and ardeparin (Figure 2B) (*N* = 5; *p* < 0.05 for each LMWH). Nifedipine did not block the effect of enoxaparin on Ca^2+^ transients (Figure 2C), showing that enoxaparin is not dependent on voltage-dependent calcium channels (*N* = 5; *p* < 0.05). Additionally, the Ca^2+^ transients induced by the CRT were reduced by enoxaparin (Figure 2D) and ardeparin (Figure 2E) (*N* = 5; *p* < 0.05 for each LMWH).

### Patch clamping

**Figure 3:**
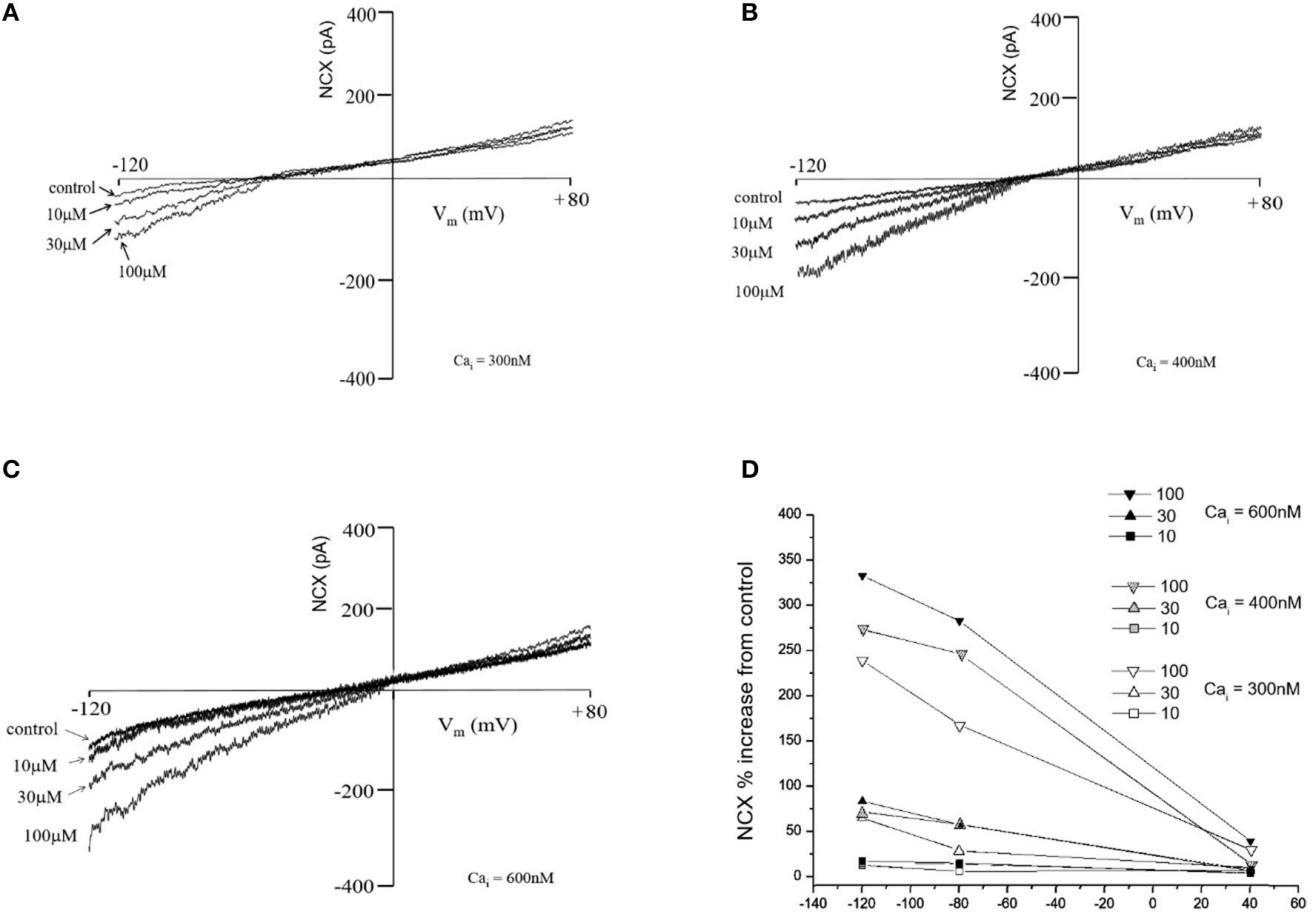
Effects of trisulfated heparin disaccharide (TD) on the NCX current (NCX) recorded from acutely isolated rat ventricular myocytes at various Ca_i_ (**A**: 300 nM; **B**: 400 nM and **C**: 600 nM). The graph **(D)** is % change obtained by dividing the current amplitude value at a given voltage for a given test compound concentration by the current value recorded in the absence of test compound (control).

Figure 3 shows the effect of TD on NCX currents. The ability of TD to activate the NCX was characterized at 3 Ca_i_ concentrations: 600, 400, and 300 nM. Such ability occurred essentially at forward mode NCX (Ca^2+^ efflux), as the NCX currents did not change above the NCX reversal potential. The effects were similar at lower Ca_i_ concentrations, although the amplitude of the forward mode NCX current was reduced.

## Discussion

In this work, we have presented experimental data that show the participation of NCX on the antiarrhythmic effect of LMWHs and trisulfated heparin disaccharide. The antiarrhythmic effects observed with these compounds were shown to both reverse or prevent arrhythmias.

The patch clamping experiments showed that the TD concentration-dependently increases the inward NCX current at Ca_i_ concentrations ranging from 300 to 600 nM. Thus, it was verified that TD can activate the forward mode NCX current (i.e., promote Ca_i_ efflux) in isolated rat cardiac myocytes. Such effect increases Ca^2+^ extrusion from the cell, as suggested by the results of calcium transient experiments. Previously, it was shown in smooth cells that TD and enoxaparin accelerate intracellular Ca^2+^ by activating the NCX (8). This indicates that the decrease in cytosolic Ca^2+^ in cardiomyocytes produced by TD or LMWHs is influenced by the enhancement of forward mode NCX activity, which is supported by the fact that the NCX mediates Ca^2+^ homeostasis similarly in many cell types (15).

Both TD and LMWHs have antiarrhythmic effects by acting in a variety of targets involved in the regulation of cardiomyocyte contraction (16). This hypothesis is corroborated by other results presented by Barry and colleagues (17), who observed that heparin disaccharides stimulate Ca^2+^ extrusion and reduce Ca^2+^ overload in pig cardiomyocytes via NCX activation and other molecule targets, such as L-type Ca^2+^ channels or Na^+^ channels. This suggests that TD and LMWHs are (at least) partially acting as an antiarrhythmic drug by reducing intracellular Ca^2+^ loading via NCX activation. As a result, we investigated a possible antiarrhythmic effect of TD and LMWHs in isolated atria and in rats *in vivo*, using experimental approaches in which Ca^2+^ overload is involved with the arrhythmogenic mechanism.

The antiarrhythmic effects of LMWHs were evaluated in isolated atria under rapid-pacing induced arrhythmia. This methodology has been used to evaluate aging and to test drugs that affect cholinergic modulation on atrial tachyarrhythmia vulnerability (9). As Ca^2+^ overload is enhanced by fast rates (18), enoxaparin and ardeparin were both evaluated as antiarrhythmic drugs to prevent atrial tachyarrhythmia. The antiarrhythmic effect of these LMWHs occurred only if NCX was fully functional (i.e., not having reverse-mode blocked by KB-R7943) indicating a crucial role of the exchanger (likely due to Ca^2+^ overload reduction) on this effect.

The *in vivo* studies show that both pre- and post-treatment with TD significantly reduced the incidence of ventricular arrhythmias and total atrioventricular block (TAVB) as well as lethality, which was zero. The influence of an ultra-low molecular weight heparin (Oligo-H, m.w. 2 kDa) on ventricular arrhythmias and lethality using equivalent *in vivo* methodology has been previously reported (11). They concluded that LMWHs significantly reduce the consequences of heart reperfusion. In the present work, we observed that the IV treatment of TD effectively prevents lethal arrhythmia and reduces animal death after arrhythmia induction.

The results show that the pre-treatment with TD (0.025 mg/kg) injected IV 15 min prior coronary occlusion significantly prevents the elevation of QT intervals induced by ischemia/reperfusion procedures. As intracellular Ca^2+^ dysregulation is a common thread of calcium-mediated arrhythmias and ECG alterations (19), it is reasonable to suggest that the effects of the TD IV treatment mitigated the Ca^2+^ overload involved with the NCX activation.

A limitation of the present study is the relatively small sample used in the experiments. However, the number of animals in each group was the minimum necessary and sufficient to achieve statistical significance shown in the results, as recommended by animal ethics committees (20).

The development of non-pharmacologic therapies to the treatment of cardiac arrhythmias, such as targeted ablation of arrhythmogenic tissues (21) and implantable cardioverter defibrillators (22), occurred mainly due to the low efficacy (between 30 and 60%) of antiarrhythmic agents in suppressing the most common arrhythmia, atrial fibrillation, and to the proarrhythmic potential of antiarrhythmic agents. However, despite the current success of ablative therapy and implantable defibrillators, the need is still pressing for new antiarrhythmic drugs (23). Indeed, antiarrhythmic drug use, such as amiodarone and lidocaine, has been preconized in patients with out-of-hospital cardiac arrest (OHCA) since bradyasystole and pulseless electrical activity (PEA), called non-shockable rhythms, may evolve to shockable ventricular fibrillation or pulseless ventricular tachycardia (VF/VT) during resuscitation in up to 25% of patients with OHCA (24, 25). Nevertheless, in amiodarone therapy, there is a higher risk of bradycardic events (26) and lack of benefit on overall mortality (27).

Thus, the potential to cause harm through pro-arrhythmic effects has placed constraints on the use of many existing antiarrhythmic drugs and restricted the release of new drugs for clinical use (28). Here, our data is showing that the new accelerator of the NCX forward mode TD efficiently suppresses Torsades de Pointes and ventricular tachycardia (VF/VT) after lethal arrhythmia induction in rats without developing major bradycardic events. Also, it is important to mention that the trisulfated heparin disaccharide, the minimum heparin fragment, has a negligible anticoagulant activity, showing that the antiarrhythmic activity of heparin oligosaccharides may be tailored for conditions in which anticoagulation therapy/effects is undesirable.

Together, our data indicate that the antiarrhythmic effects of TD and LMWHs are related to intracellular Ca^2+^ overload attenuation by the NCX acceleration. Thus, as the stimulation of the forward mode NCX activity reduces cytosolic Ca^2+^ and exert a therapeutic action (29, 30), the data provide strong evidence that the heparin oligosaccharides, even those with very low anticoagulant activity as depicted by TD, may constitute a new class of antiarrhythmic drug that acts mechanistically by accelerating the NCX forward mode.

## Author contributions

CG: conception and definition of all experiments procedures; patch clamping; *in vitro* and *in vitro* heparin oligosaccharides experimental tests; all data analysis; drafting and revising the work; IT and HN: conception and definition of all experiments procedures; production of heparin oligosaccharides; all data analysis; revising the work; ÊV: conception and definition of experimental procedures related to in vitro and in vitro experiments; development and data analyses of experimental tests of heparin oligosaccharides (*in vivo* and *in vitro*); revising the work; ML: production of heparin oligosaccharides; data analysis; revising the work; AC and RM-F: development and revision of Calcium transient experimental tests; data analysis; JT, BA, and JD: Development of experimental tests of heparin oligosaccharides (*in vivo* and *in vitro*); data analysis.

### Conflict of interest statement

CG, IT, and EV received grants from Sanofi-Aventis. The remaining authors declare that the research was conducted in the absence of any commercial or financial relationships that could be construed as a potential conflict of interest.
